# Gut Health in Veterinary Medicine: A Bibliometric Analysis of the Literature

**DOI:** 10.3390/ani11071997

**Published:** 2021-07-03

**Authors:** Elena Colombino, Daniel Prieto-Botella, Maria Teresa Capucchio

**Affiliations:** 1 Department of Veterinary Sciences, University of Turin, 10095 Grugliasco, Italy; mariateresa.capucchio@unito.it; 2 Department of Surgery and Pathology, Miguel Hernandez University, 03550 Alicante, Spain; dprieto@umh.es

**Keywords:** gut health, veterinary medicine, bibliometric analysis

## Abstract

**Simple Summary:**

Gut health has been a main topic in veterinary medicine research after the ban on the use of antimicrobial growth promoters. Gut health has been defined as absence/prevention/avoidance of gastrointestinal disease so that the animal is able to perform its physiological functions. A bibliometric analysis is a set of statistical methods used to explore trends in the scientific literature such as number of publications, most prolific countries and main research areas to highlight publication dynamics and gaps of knowledge. In this case, a bibliometric analysis was performed on veterinary gut health using the database Web of Science and the R package Bibliometrix. A total of 1696 documents were retrieved between 2000 and 2020, showing an increase of 22.4% in the number of annual publications. Pigs (34.8%), poultry (chicken, duck, turkey and quail—33.9%) and aquaculture (fishes, crustaceans and frog—15.0%) were the most studied species while a scarce number of publications was found on felines, cows, horses, rodents, goats and sheep. China (24.7%), USA (17.2%) and Canada (5.7%) were the most productive countries. Three main research lines aimed to explore animal nutrition, prevention of inflammatory diseases and microbiota composition were identified. This study will help drive future research on the topic.

**Abstract:**

Gut health is a recent relevant research topic in veterinary medicine and it has been shown to be associated with better zootechnical performances and animal welfare. A bibliometric analysis was performed to explore publication trends, dynamics and possible knowledge gaps in this field in the last twenty years (2000–2020). The database Web of Science was searched and the results were analyzed by the R package Bibliometrix. A total of 1696 documents were retrieved in the evaluated period, showing a constant annual growth in the number of publications of 22.4%. These articles focused mainly on pigs (34.8%), poultry (chicken, duck, turkey and quail—33.9%) and aquaculture (fishes, crustaceans and frog—15.0%) while a scarce number was found on felines, cows, horses, rodents, goats and sheep. China was the most productive country (24.7%) followed by the USA (17.2%) and Canada (5.7%). Keyword’s analysis showed that the main research lines aimed to explore animal nutrition, prevention of inflammatory diseases and microbiota composition. This study shows a comprehensive bibliometric analysis of the evolution of veterinary gut health research that will help to drive future investigations on this topic.

## 1. Introduction

In recent decades, the term “gut health” has become increasingly popular and frequently used in the scientific literature regarding human and veterinary medicine [1,2]. A consensus on the definition of “gut health” has not yet been reached as the intestine is a complex organ with digestive, immunological, neurological and endocrine functions [3]. Gut health is generally defined as the absence, prevention or avoidance of intestinal disease so that the animal is able to perform its physiological functions in order to withstand exogenous and endogenous stressors [2]. However, a broader definition of gut health should cover multiple positive aspects of the gastrointestinal tract including effective digestion and absorption of feed, the proper structure of gastrointestinal barrier, the absence of gastrointestinal illness, normal and stable intestinal microbiota, effective immune status, and proper control of the enteric nervous system [1].

Gut health relies on the maintenance of a delicate balance between the host, the intestinal environment and the dietary compounds [3]. Recently, it has also been shown that there is an extensive communication between the brain and the microbiota via the brain-gut-microbiome axis. Through this bidirectional communication, signals from the brain can influence the motor/sensory/secretory functions of the gut, and visceral messages from the gut can influence brain function [4]. On the one hand, in food producing animals, gut health can be considered a synonymous of animal health, strictly linked to animals’ growth performances. In fact, if gut health is compromised, digestion and nutrient absorption are affected with a detrimental effect on feed conversion ratio leading to economic loss and a greater susceptibility to disease [3]. On the other, in companion animals a healthy gut is crucial for their well-being and changes in gut microbiota have already been related to a multitude of disorders such as inflammatory bowel disease (IBD) but also cardiovascular disease and allergies [5,6]. Moreover, fecal microbiome transplant has been studied as a treatment option for multiple gastrointestinal diseases, such as IBD [7].

Gut health can be significantly affected by factors such as animal’s management, feed quality and environment [3]. To date, a large number of studies have proved that diet is the most influential factor on gut health [3,8,9]. In fact, innovative feed ingredients, probiotics and prebiotics could positively modulate gut microbiota [10], gut barrier function [8,9] and mucin composition [11] with a significant reduction in disease incidence both in pets and food-producing animals. Moreover, stress has been proven to have a negative effect on gut health impairing especially gut microbiota and causing dysbiosis, which is a disruption of the microbiota composition accompanying intestinal inflammation [12].

To date, modulation of gut health could play a key role in reducing the need of antimicrobials and in protecting animals from diseases, which is particularly relevant considering the ban on the use of antimicrobial growth promoters imposed by the European Union in 2006 and the reassessment of their use in the USA [13]. Finding alternatives to antibiotics for mantaining gut health as well as systemic health in animals is even more important in a concept of One Health in order to help preserving the effectiveness of antimicrobials that are important for human medicine by reducing their use in animals [14].

Despite the high number of published articles and reviews on the topic, the evolution of the research in gut health still remains largely unknown. Bibliometric analysis represents an interesting approach to analyze a large amount of publication in order to investigate dynamics of research literature production, study the impact of journals, determine citation patterns, and identify research themes and future directions or hot topics [15]. In veterinary medicine only a few bibliometric studies have been published on animal welfare, large animal’s interleukins and organic livestock production [16,17,18]. To the author’s knowledge, no previous bibliometric analysis on gut health has been conducted so far.

Therefore, the aim of the present study was to perform a bibliometric analysis on gut health research in veterinary medicine to evaluate the current trends, the presence of gaps of knowledge and the future perspective on the topic.

## 2. Material and Methods

### 2.1. Search Strategy and Data Acquisition

In this bibliometric study, publications on veterinary gut health were retrieved from the Web of Science (WoS) database on 19 February 2021. Specifically, WoS Core Collection was used due to the rigorous selection and evaluation process of the reported academic information [19]. The search equation was developed by using the main gut health and animal species terms identified in the literature as follows: (“GUT HEALTH” OR “INTESTINAL HEALTH”) AND (“POULTRY” OR “PIG” OR “FISH” OR “COW” OR “HORSE” OR “SHEEP” OR “DUCK” OR “RABBIT” OR “GOAT” OR “CAT” OR “DOG”). In addition, the search was performed by WoS topic field that includes title, abstract and author keywords and a 2000–2020 timespan. Raw data was extracted in BibTeX and txt formats using the WoS extraction tool. Information fields related to authors, affiliations, journals, keywords, research areas, citations, titles and abstracts were included in the extraction. In order to minimize any mistakes or missing information and to identify the animal species analyzed in each document, a manual revision of the articles was performed by EC and DP-B. Animal species were categorized as follows: Pig, Poultry (chicken, duck, turkey and quail), Aquaculture (fishes, crustaceans and frog), Rabbit and Rodent (mouse and rat), Cat and Feline (lynx and tiger), Cow, Horse, Goat and Sheep.

### 2.2. Data Analysis

The bibliometric analysis of the WoS raw data was conducted using R software version 4.0.4 (R Foundation for Statistical Computing, Vienna, Austria;). Concretely, the analysis was performed by Bibliometrix R package version 3.0.4 [20]. This package includes all the main bibliometric methods to measure time trends, identify the most cited papers and detect the most prolific authors, journals, institutions and countries. In particular, author contribution was described with H-index (number of papers N that have N or more citations), G-index (H-index variant where papers with more citations are weighted) and M-index (H-index divided by number of years in doing active research). In addition, Bibliometrix provides mapping analysis to visualize relevant information such as keyword co-occurrences network maps. To complete the bibliometric results, the impact factors (IF) of the yielded journals were extracted from the latest Journal Citation Reports (JCR, 2019) by Clarivate Analytics. Information regarding research areas and type of document was categorized using the WoS results analysis function.

## 3. Results

### 3.1. General Research Outputs

**Figure 1:**
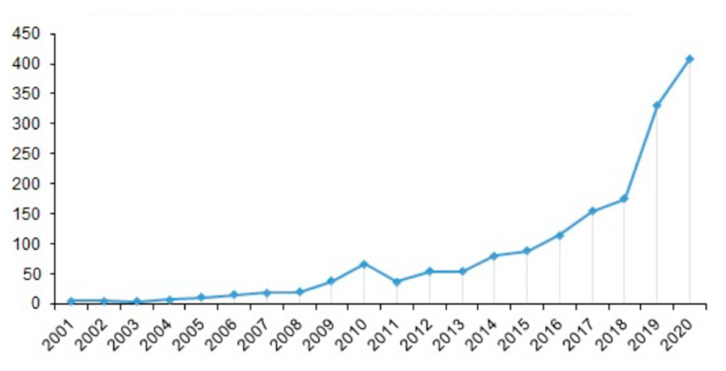
Timespan of publications on gut health in animals.

In total, 1696 documents were published between 2000 and 2020 on veterinary gut health research. The annual evolution of publications during this time span is reported in Figure 1. The annual growth rate was 22.4%, with a mean of 4.6 articles per year. Particularly, the production increased exponentially from 2011 to 2020, being 2020 the most prolific year with 417 published articles. In addition, the production increased substantially between 2018 and 2019, changing from 175 to 315 articles, respectively. Regarding document type, the majority of retrieved publications were research articles (n = 1427, 84.1%) followed by reviews (n = 163, 9.6%), meeting abstracts (n = 98, 5.8%), editorials (n = 6, 0.4%) and retractions (n = 2, 0.1%).

### 3.2. Research Areas

**Figure 2:**
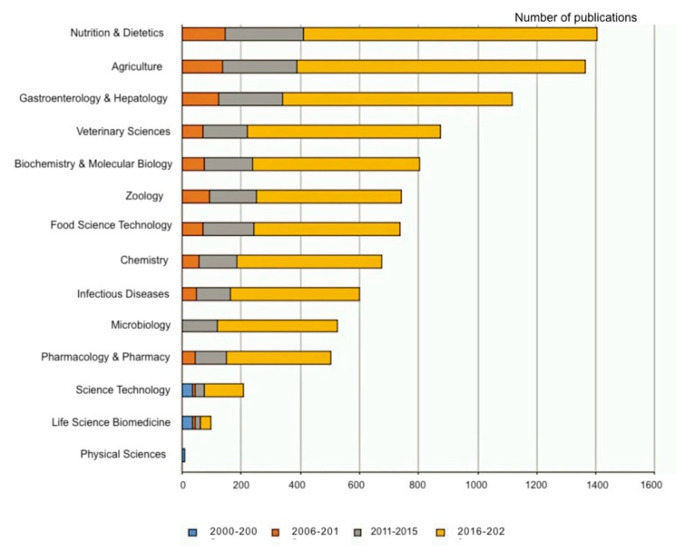
Distribution of publications among the most studied research areas.

Figure 2 reported the distribution of the retrieved documents by research areas according to WoS. In the period 2000–2005, the yielded documents were organized in three main categories: Science technology, Life Science Biomedicine and Physical Science. Due to the addition of more detailed subject areas in WoS in recent periods (2006–2010; 2011–2015 and 2016–2020) the majority of publications were included in “Nutrition & Dietetics” (n = 1405, 82.8%), “Agriculture” (n = 1362, 80.3%), “Gastroenterology & Hepatology” (n = 1118, 65.9%), “Veterinary Sciences” (n = 876, 51.7%), and “Biochemistry & Molecular Biology” (n = 801, 47.2%). Furthermore, in the period 2011–2015, “Microbiology” appeared among the research areas studied in veterinary gut health (n = 120, 7.1%).

### 3.3. Publication Analysis Based on Species

**Figure 3:**
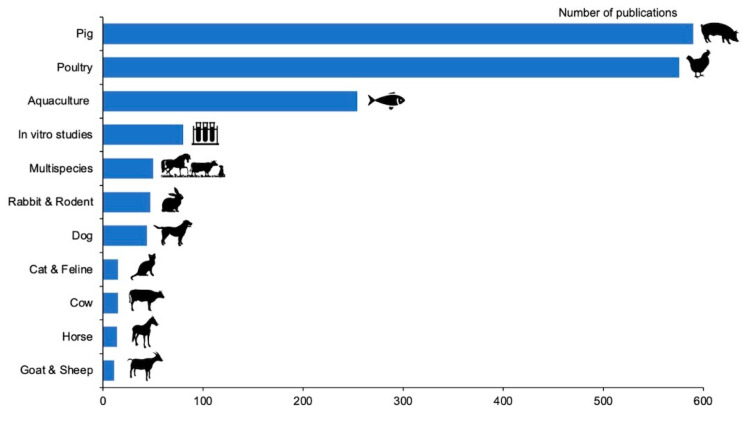
Distribution of publications based on animal species.

A total of 11 animal categories were identified among the retrieved documents and their distribution can be observed in Figure 3 Pig, (n = 590, 34.8%), poultry (n = 576, 33.9%) and aquaculture (n = 254, 15.0%) were the most studied ones, representing the 83.7% of the whole research on gut health. On the contrary, cat and feline, cow, horses, goat and sheep were the less studied animal categories, totaling 3.2% (n = 55) of the whole gut health research. Moreover, in vitro (n = 80, 4.7%), multispecies (n = 50, 2.9%), rabbit and rodents (n = 47, 2.8%) and dog (n = 44, 2.7%) studies were also detected.

### 3.4. Key Journals Related to Veterinary Gut Health Research

Three hundred and seventeen journals have published articles related to gut health research in veterinary medicine. In Table 1, the 20 most prolific journals are reported. As the majority of retrieved documents were original articles, journals were considered the main source for publication. A total of 906 documents were published in the top 20 most prolific journals, accounting for 53.4% of the total publications in veterinary gut health (n = 1696). In particular, *Journal of Animal Science*, *Poultry Science*, *Animals* and *Aquaculture* showed the highest number of articles with 150 (8.9%), 145 (8.6%), 67 (4.0%) and 56 (3.3%) documents, respectively. Thirteen (65.0%) of the top-20 most prolific journals were mainly focused on “Agriculture, dairy & Animal science”. Regarding journal relevance, seven journals among the most prolific ones had an IF greater than 3, *Animal Nutrition* being the first in rank (IF = 4.492). These seven most relevant journals collected 230 papers, representing the 13.6% of the total scientific literature on gut health (n = 1696).

### 3.5. Authors’ Publication Performance

A total of 5664 authors were involved in the authorship of the retrieved documents (n = 1696), with a mean of 3.3 authors per document and 0.3 documents/author. In particular, most of the articles were multi-authored (n = 1644) while 52 articles were single-authored. Moreover, 4208 authors published only one article, representing the 74.3% of all authors. Among the authors who published more than one article, 1255 published between 2 and 5 documents, 141 between 6 and 10, 47 between 11 and 20 and 13 more than 20. In addition, a collaboration index of 3.4 was calculated through a co-authorship analysis as the total numbers of authors of multi-authored papers divided by the total number of multi-authored articles. Therefore, a mean of 5.6 co-authors per document was identified. The most prolific authors in gut health research (≥30 articles) were Li, Y. and Liu, Y. with a total of 38 and 36 published papers, respectively (Table 2).

### 3.6. Countries and Affiliation Analysis

Considering the corresponding author’s affiliation, the selected documents were from 53 countries over 5 continents. Table 3 displays general information regarding the 20 most prolific countries in veterinary gut health research. The top-20 most prolific countries accounted for 89.6% (n = 1519) of total published articles in gut health (n = 1696). The most productive country was China that published almost a quarter of the scientific production (n = 419, 24.7%), followed by the USA (n = 292, 17.2%) and Canada (n = 96, 5.7%). Regarding inter-country collaboration, the number of multiple country publications (MCP) was extracted and the MCP ratio (MPC articles/total publications per country) was then calculated. The country showing the higher proportion of MCP was Egypt (66.7%, MCP ratio = 0.7) followed by France (56.7%, MCP ratio = 0.6), Australia (56.5%, MCP ratio = 0.6), Belgium (50.0%, MCP ratio = 0.5) and the Netherlands (45.1%, MCP ratio = 0.4). On the contrary, Brazil (84.6%), Ireland (83.8%), Iran (79.3%), China (77.1%) and USA (77.1%) showed the highest rate of intra-country collaboration with a greater proportion of single-country publications (SPC).

Authors of the 1696 retrieved documents were from 1412 institutions. Information regarding the top 20 most prolific research institutions is summarized in Table 4. These institutions published 461 articles, corresponding to 27.2% of the total scientific literature on gut health in animals. Geographically, seven of the 20 most prolific institutions were from China (35.0%), being the most represented country followed by the USA (n = 4, 20.0%) and Canada (n = 2, 10.0%). Similarly, China and the USA also hosted the top three most productive institutions, which are the China Agricultural University (n = 57, 3.4%), the Sichuan Agricultural University (n = 49, 2.9%) and the North Carolina State University (n = 40, 2.4%).

### 3.7. Citation Analysis and Most Relevant Papers

Data from citation indexes were analyzed to determine the popularity and impact of publications on gut health in veterinary science. A total of 28,221 citations were obtained from the 1696 retrieved documents (16.6 citations per document). The majority of papers showed between 1 and 10 citations (n = 757, 44.6%). The remaining 939 papers were distributed as follows: 356 papers (20.9%) had no citations, 347 (20.5%) between 11 and 30, 96 (5.7%) between 31 and 50, 88 (5.2%) between 51 and 100 and 52 (3.1%) had more than 100 citations.

The top 20 most productive authors reported in Table 2 accounted for 6527 citations, representing the 23.1% of the total. Among them, the most cited author was Van Immersel F. with 743 citations, followed by Ducatelle R. with 654 and Wang L. with 639 citations, respectively. Conversely, authors with the highest number of citations were not the ones with the highest H-index. In fact, the author with the highest H-index was Li Y. with an H-index of 13, followed by Liu Y and Wang L. with an H-index of 11. Considering the first publication year of each author, the m-index showed that Li X. (1.4), Li Y. (1.3) and Zhang X. (1.2) were the ones who showed the greater growth in their scientific production.

The top 20 most cited papers are listed in Table 5 and they encompass 4893 citations (17.3% of the total). They were published in 15 different scientific journals. Particularly, *Poultry Science* accounted for three of the top cited articles (Awad et al., 2009; Yegani et al., 2008 and Baurhoo et al., 2007). The most cited paper was “A review of interactions between dietary fibre and the intestinal mucosa, and their consequences on digestive health in young non-ruminant animals” written by Montagne, L. et al. in 2003 and published in Animal Feed Science and Technology (IF: 2.582), receiving 511 citations with an average of 63.8 citations per year. None of the top-20 most cited and productive authors was listed among the authors of the most cited papers.

Regarding countries, the top-20 most productive countries reported in Table 3 accounted for 25658 citations, which is the 90.1% of the total citations recorded for veterinary gut health articles. In particular, China was the most cited country (n = 4853, 17.2%) followed by the USA (n = 4553, 16.1%) and Canada (n = 2440, 8.6%).

### 3.8. Keywords Co-Occurrence Analysis

**Figure 4:**
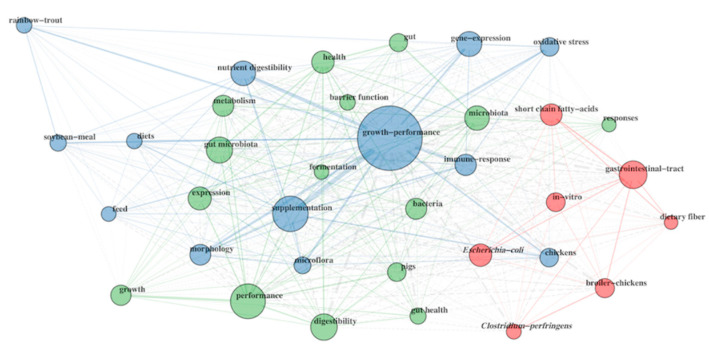
Keyword plus co-occurrence network map.

A total of 3111 author keywords were obtained from the 1696 retrieved documents, being gut health (n = 251, 8.1%), intestinal health (n = 166, 5.3%), microbiota (n = 136, 4.4%), growth performances (n = 132, 4.2%) and broiler (n = 127, 4.1%) the most frequently used terms. Moreover, 3817 keywords plus were also retrieved as they enhanced the power of cited-reference searching thanks to a special algorithm that is unique to Clarivate Analytics databases. In particular, growth performance (n = 433, 11.3%) was the most recurrent keyword plus followed by supplementation (n = 214, 5.6%), performance (n = 205, 5.4%), digestibility (n = 138, 3.6%) and gastrointestinal tract (n = 133, 3.5%). The co-occurrences analysis of the 35 most frequent keywords Plus can be observed in Figure 4. In this figure, the nodes diameter represents the keyword frequency while the thickness of the path lines represents the co-occurrence relationships. In the present study, network analysis of the keyword plus revealed three clusters. Particularly, the blue cluster was composed by the following keywords: rainbow trout, chickens, soybean meal, diets, feed, nutrient digestibility, morphology, supplementation, microflora, gene expression, immune response, oxidative stress and growth-performance. The green cluster included pigs, gut microbiota, metabolism, expression, growth, performance, digestibility, fermentation, barrier function, health, gut, microbiota, bacteria, gut health and responses. Finally, the red cluster included broiler chickens, short-chain fatty acids, in vitro, Escherichia coli, Clostridium perfringens, dietary fiber, gastrointestinal tract.

## 4. Discussion

The aim of this study was to evaluate through a bibliometric analysis the current publication trends and dynamics in veterinary gut health research. The analysis showed an increased number of publications in veterinary gut health in the last decade. The majority of documents were published in China and USA between 2011 and 2020, being primarily research articles. The yielded documents mainly focused on poultry, pigs and aquaculture and the most discussed research topics were linked to nutrition and dietetics.

The evaluation of publications time span in the last 21 years (2000–2020) showed that gut health is an emerging research topic. In fact, the annual number of publications has been constantly increased, reaching its high in 2020 and reflecting an overall steady improvement. It could be hypothesized that the growing interest in gut health followed the European Union ban on antimicrobial growth promoters (AGP) in animal feed in 2006 as gut health-related problems became an important issue in intensive animal farming [21]. Thus, many researchers at the beginning of the XXI century focused their research work in finding valuable alternatives to antibiotics that could positively modulate gut health, boosting this new research topic [22].

The majority of publications were research articles (n = 1427, 84.1%) mainly focused on “Nutrition & Dietetics” (n = 1405, 82.8%), “Agriculture” (n = 1362, 80.3%), “Gastroenterology & Hepatology” (n = 1118, 65.9%), “Veterinary Sciences” (n = 876, 51.7%), and “Biochemistry & Molecular Biology” (n = 801, 47.2%). This could be explained by the fact that gut health has been demonstrated to be mainly influenced by diet [3]. Thus, the veterinary gut health research focused on testing the effect of different feed ingredients or additives on multiple gut parameters. Moreover, in recent years, “Microbiology” gained importance among the research areas studied in veterinary gut health (n = 120, 7.1%). Current research has recognized that the composition of gut microbiota or the microbiome is one of the key factors in maintaining gut health as it is involved in nutrient absorption, feed digestibility, energy harvest and therefore animals’ productivity [23]. In addition, the development of next-generation sequencing techniques and biomolecular techniques helped in having a deeper insight into the microbiological aspects of gut health [24]. The presence of different research areas also demonstrated that a multidisciplinary approach is needed for an exhaustive evaluation of veterinary gut health [21].

Regarding animal species, most of the studies focused on pigs (34.8%), poultry (33.9%) and aquaculture (15.0%). This is probably due to the fact that poultry and pigs are ones of the most ubiquitous livestock species worldwide with almost 19.60 billion chickens and 0.98 billion pigs in the world [25]. Moreover, poultry, and pigs were the main livestock sectors that used AGP and they were more affected by the ban imposed by the EU in 2006, requiring valuable alternatives [21,26]. Aquaculture represents the main source of valuable animal protein worldwide and it attracted increasing attention due to the decline of capture fisheries, becoming the fastest growing food production animal worldwide [27]. Moreover, a greater interest in improving these rearing systems and optimizing animal productive performances through the modulation of gut health has grown in response to increasing demand for animal-based protein for human consumption [28]. However, a lack of knowledge in ruminants (cows, sheep and goats), horses, rabbits, cats and dogs was detected. A possible explanation for this gap is the difficulty in conducting research in these animals, particularly ruminants and pets. On the one hand, ruminants have a longer production cycle and require bigger spaces for their rearing compared to other species, making them difficult to use in research [29]. On the other, companion animals are more difficult to enroll for clinical trials or research works and non-invasive procedures must be preferred, representing a potential limitation for researchers.

This trend is also confirmed by the appearance of rainbow trout, pigs and chickens among the 35 most cited keywords plus. Moreover, keyword’s network highlighted three main areas of interest: (i) one related to animal nutrition and zootechnical parameters; (ii) one related to immunology, gene expression and oxidative stress and (iii) one related to microbiology and infectious disease. This revealed that the main research lines are aimed to test different dietary feed ingredients, improve animals’ productivity, prevent gastrointestinal diseases and drive microbiota composition [30]. However, it can be pointed out that some innovative concepts such as gut–brain axis and fecal transplants were not detected among the most frequent keywords, suggesting that they probably need to be developed in the next future. In fact, the gut–brain axis seems to influence the host neural function and behavior, particularly those relevant to stress-related disorders. Thus, regulating the gut microbiome could help improving animal welfare [31]. Moreover, fecal transplants has been explored as a treatment for IBD in dogs but whether it is an effective and safe option for canine IBD still remains unknown [32].

The top-3 most prolific journals were *Journal of Animal Science* (8.9%), *Poultry Science* (8.6%) and *Animals* (4.0%). These are all English language journals and they are all included in the first quartile for *Agriculture, Dairy and Animal Science* according to 2019 JCR. *Journal of Animal Science* and *Animals* encompass a broad range of research topics in animal production and fundamental aspects of genetics, nutrition, physiology, preparation and utilization of animal products. On the contrary, *Poultry Science* is the highest-ranked (by Impact Factor) journal dedicated to publishing poultry research and it also account for three of the top-20 cited documents on veterinary gut health (Baurhoo et al., 2007; Yegani et al., 2008 and Awad et al., 2009). Interestingly, the fourth most prolific journal is Aquaculture which is one of the top-ranked journals in “Marine and freshwater biology” according to JCR. This is in accordance with the three top-studied species and with the keyword’s analysis.

Geographical distribution of publication on veterinary gut health is mainly located in China (24.7% of total publication) and USA (17.2%). Accordingly, the three most productive institutions were also from China (China Agricultural University and Sichuan Agricultural University) and USA (North Carolina State University). This pattern is far from being restricted to veterinary gut health. In fact, USA and China were the most productive countries in Agricultural and Biological Science according to Scimago Journal and Country Rank (https://www.scimagojr.com/countryrank.php, Access date: 19 February 2021) and they were also the first countries for poultry, pig and fish production systems [28].

Considering the author’s metrics, the majority of the top-20 most prolific authors started to publish in 2010 and the m-index showed that they have had a high scientific production in a relatively short period of time. Thus, this corroborates that gut health is a relatively novel research topic and it is in accordance with the greater growth of research on veterinary gut health observed from 2011. Moreover, the great numbers of citations achieved by the top-20 most prolific authors and by the 20-most cited papers in this short period of time reflects the growing interest of the scientific community on the topic.

This study has several limitations. Firstly, the search was conducted solely in WoS, thus articles and journals not listed in this database have not been included in the results with a possible underestimation of them. Nevertheless, WoS is a long and well-established database characterized by a wide range of scientific journals [19]. Secondly, in the search equation some of the species were grouped (e.g., poultry and fish) and this can lead to possible inclusion bias. However, an exhaustive search including the main species was conducted. Thirdly, WoS and Bibliometrix were used for data extraction and transformation. These procedures can provoke misleading results or missing data. Therefore, the bibliographic information was independently revised by EC and DP-B. Despite these limitations, to the author’s knowledge this is the first bibliometric analysis addressing veterinary gut health. Furthermore, this study offers insightful data on the research areas, animal species, main contributors and publication’s performances on veterinary gut health. Finally, this study can help to detect potential gaps of knowledge and address future research on gut health.

## 5. Conclusions

This study showed that gut health is a relevant research area in veterinary medicine with a constant increment in publication from 2010 to present. The current research mainly focuses on pigs, poultry and aquaculture with three main lines of research: nutrition, immunology and microbiology. An important gap of knowledge was also detected regarding research on other species, mainly ruminants, horses, rabbits, cats and dogs. In conclusion, future research could focus on the evaluation of gut health in the abovementioned less investigated species in order to explore its main component (animal nutrition, zootechnical parameters, immunology, gene expression, oxidative stress and microbiota) that have been already explored in pigs, poultry and aquaculture. Regardless of the species, future investigations should deepen in novel areas such as the evaluation of gut–brain axis and its function or the potential of fecal microbiota transplants as a treatment for gastrointestinal diseases.

## Figures and Tables

**Table 1:** Top 20 most prolific journals on veterinary gut health research sorted by number of publications.

Journal	Number of Publications (% ^1^)	Category (Rank)	2019 JCR IF
*Journal of Animal Science*	150 (8.9)	Agriculture, Dairy and Animal Science (12/63)	2.092
*Poultry Science*	145 (8.6)	Agriculture, Dairy and Animal Science (7/63)	2.659
*Animals*	67 (4.0)	Agriculture, Dairy and Animal Science (7/63); Veterinary Sciences (14/141)	2.323
*Aquaculture*	56 (3.3)	Fisheries (5/53); Marine and Freshwaters Biology (11/107)	3.225
*Animal Feed Science and Technology*	55 (3.20)	Agriculture, Dairy and Animal Science (8/63)	2.582
*Fish and Shellfish immunology*	46 (2.7)	Fisheries (6/52); Immunology (76/158); Marine and Freshwaters Biology (12/108); Veterinary Sciences (3/141)	3.298 ^2^
*Livestock Science*	46 (2.7)	Agriculture, Dairy and Animal Science (18/63)	1.700
*Journal of Animal Physiology and Animal Nutrition*	37 (2.2)	Agriculture, Dairy and Animal Science (25/63); Veterinary Sciences (42/141)	1.597
*Animal*	34 (2.0)	Agriculture, Dairy and Animal Science (9/63); Veterinary Sciences (11/141)	2.400
*Frontiers in Microbiology*	33 (1.9)	Microbiology (34/136)	4.236
*PLoS ONE*	32 (1.9)	Multidisciplinary Sciences (27/71)	2.740
*Journal of Animal Science And Biotechnology*	29 (1.7)	Agriculture, Dairy and Animal Science (3/63)	4.167
*Frontiers in Veterinary Science*	27 (1.6)	Veterinary Sciences (19/141)	2.245
*Worlds Poultry Science Journal*	25 (1.5)	Agriculture, Dairy and Animal Science (15/63)	1.802
*British Journal of Nutrition*	24 (1.4)	Nutrition and Dietetics (40/89)	3.334
*Aquaculture Nutrition*	22 (1.3)	Fisheries (16/53)	2.231
*Animal Nutrition*	21 (1.2)	Agriculture, Dairy and Animal Science (2/63); Veterinary Sciences (3/141)	4.492
*Journal of Dairy Science*	21 (1.2)	Agriculture, Dairy and Animal Science (5/63), Food Science and Technology (37/139)	3.333
*British Poultry Science*	19 (1.1)	Agriculture, Dairy and Animal Science (27/63)	1.537
*Animal Production Science*	17 (1.0)	Agriculture, Dairy and Animal Science (36/63)	1.215

JCR: Journal Citation Reports; IF: Impact Factor. ^1^ Over 1696. ^2^ 2018 Impact Factor.

**Table 2:** Top 20 cited authors on veterinary gut health research.

Author	H Index	G Index	M Index	TC	NP	PY Start
Li, Y.	13	20	1.3	445	38	2011
Liu, Y.	11	18	1.1	379	36	2011
Wang, L.	11	24	0.9	639	24	2009
Sweeney, T.	10	20	0.6	446	26	2005
O’Doherty, JV.	10	22	0.9	504	23	2005
Yu, B.	9	15	0.2	251	26	2007
Van Inmerseel, F.	9	19	0.8	743	19	2009
Kiarie, E.	9	18	0.8	411	18	2010
Wang, Y.	8	15	1.1	239	25	2014
Li, J.	8	10	0.5	127	23	2006
Chen, D.	8	11	1.1	155	23	2014
He, J.	8	15	1.1	244	23	2014
Mao, X.	8	13	0.7	181	20	2010
Yin, Y.	8	18	0.7	399	18	2010
Ducatelle, R.	8	17	0.7	654	17	2010
Li, X.	7	9	1.4	111	23	2016
Wang, J.	7	9	1.0	96	19	2014
Zhang, Y.	6	12	0.9	165	24	2014
Zhang, X.	6	8	1.2	87	17	2016
Kim, SW.	4	15	0.3	251	23	2009

TC: Total Citations; NP: Number of Publications; PY: Publication Year.

**Table 3:** Top 20 most prolific countries on veterinary gut health research sorted by publication number.

Country	Publications Number	% ^1^	SCP	MCP	MCP Ratio ^2^	TC
China	419	24.7	323	96	0.2	4853
USA	292	17.2	225	70	0.2	4553
Canada	96	5.7	58	38	0.4	2440
Brazil	78	4.6	66	12	0.2	306
Australia	62	3.7	27	35	0.6	1723
Spain	58	3.4	33	25	0.4	1032
United Kingdom	55	3.2	31	24	0.4	1672
Belgium	54	3.2	27	27	0.5	886
Italy	51	3.0	33	18	0.4	517
Netherlands	51	3.0	28	23	0.4	1388
Norway	40	2.4	25	15	0.4	1154
South Korea	38	2.2	26	12	0.3	314
Ireland	37	2.2	31	6	0.2	657
India	33	1.9	26	7	0.2	567
France	30	1.8	13	17	0.6	2168
Germany	30	1.8	20	10	0.3	508
Iran	29	1.7	23	6	0..2	261
Denmark	25	1.5	17	8	0.3	383
Egypt	21	1.2	7	14	0.7	157
Poland	20	1.2	15	5	0.3	119

SCP: Single Country Publications; MCP: Multiple Country Publications; TC: Total Citations. ^1^ Over 1696. ^2^ Expressed as the MPC articles divided by the total publications per country.

**Table 4:** Top 20 most productive research institutes sorted by number of publications.

Research Institute	Number of Publications	% ^1^	Country
China Agricultural University	57	3.4	China
Sichuan Agricultural University	49	2.9	China
North Carolina State University	40	2.4	USA
University of Illinois	31	1.8	USA
Ghent University	30	1.8	Belgium
University College Dublin	26	1.5	Ireland
Nanjing Agricultural University	25	1.5	China
The University of Manitoba	23	1.4	Canada
The University of Guelph	19	1.1	Canada
University of New England	19	1.1	USA
Universidad Autónoma de Barcelona	17	1.0	Spain
Ocean University of China	15	0.9	China
The University of Georgia	15	0.9	USA
Wageningen University	15	0.9	Netherlands
Dankook University	14	0.8	South Korea
Institute of Animal Science	14	0.8	Czech Republic
Institute of Subtropical Agriculture	14	0.8	China
University of Bologna	14	0.8	Italy
Huazhong Agricultural University	12	0.7	China
Northwest A&F University	12	0.7	China

^1^ Over 1696.

**Table 5:** Top 20 most cited articles on veterinary gut health.

Title	Author	Year	Journal	IF ^1^	TC
A review of interactions between dietary fibre and the intestinal mucosa, and their consequences on digestive health in young non-ruminant animals	Montagne, L. et al.	2003	*Animal Feed Science and Technology*	2.582	511
Effects of dietary inclusion of probiotic and symbiotic on growth performance, organ weights, and intestinal histomorphology of broiler chickens	Awad, WA. et al.	2009	*Poultry Science*	2.659	356
From the gut to the peripheral tissues: the multiple effects of butyrate	Guilloteau, P. et al.	2010	*Nutrition Research Reviews*	7.641	322
BOARD-INVITED REVIEW: opportunities and challenges in using exogenous enzymes to improve nonruminant animal production	Adeola, O. and Coweison, AJ.	2011	*Journal of Animal Science*	2.092	295
Fermentation in the large intestine of single-stomached animals and its relationship to animal health	Williams, Ba. et al.	2001	*Nutrition Research Reviews*	7.641	295
Important antinutrients in plant feedstuffs for aquaculture: an update on recent findings regarding responses in salmonids	Krogdahl, A. et al.	2010	*Aquaculture Research*	1.748	282
The effect of herbs and their associated essential oils on performance, dietary digestibility and gut microflora in chickens from 7 to 28 days of age	Cross, DE. et al.	2007	*British Poultry Science*	1.537	274
Nutritional management of gut health in pigs around weaning	Lalles, JP. et al.	2007	*Proceedings of the nutrition society*	5.577	247
Genomic characterization of the uncultured Bacteroidales family S24-7 inhabiting the guts of homeothermic animals	Ormerod, KL. et al.	2016	*Microbiome*	11.607	220
Factors affecting intestinal health in poultry	Yegani, M. and Korver, DR.	2008	*Poultry Science*	2.659	215
Effects of purified lignin and mannan oligosaccharides on intestinal integrity and microbial populations in the ceca and litter of broiler chickens	Baurhoo, B. et al.	2007	*Poultry Science*	2.659	212
The chicken gastrointestinal microbiome	Oakley, BB. et al.	2014	*FEMS Microbiology Letters*	1.987	207
Microbiota of the chicken gastrointestinal tract: influence on health, productivity and disease	Stanley, D. et al.	2014	*Applied Microbiology and Biotechnology*	3.530	197
Supplemental fructooligosaccharides and mannanoligosaccharides influence immune function, ileal and total tract nutrient digestibilities, microbial populations and concentrations of protein catabolites in the large bowel of dogs	Swanson, KS. et al.	2002	*The Journal of Nutrition*	4.281	188
The fecal microbiome in dogs with acute diarrhea and idiopathic inflammatory bowel disease	Suchodolski, JS. et al.	2012	*PLoS ONE*	2.740	187
Performance responses and indicators of gastrointestinal health in early-weaned pigs fed low-protein amino acid-supplemented diets	Nyachoti, CM. et al.	2006	*Journal of Animal Science*	2.092	181
Bacteria, phages and pigs: the effects of in-feed antibiotics on the microbiome at different gut locations	Looft, T. et al.	2014	*The ISME Journal*	9.180	180
Strategic use of feed ingredients and feed additives to stimulate gut health and development in young pigs	De Lange CFM. et al.	2010	*Livestock Science*	1.700	179
Managing gut health through nutrition	Choct, M.	2009	*British Poultry Science*	1.537	173
Feed particle size: Implications on the digestion and performance of poultry	Amerah, AM. et al.	2007	*Worlds Poultry Science Journal*	1.802	172

IF: Impact Factor; TC: Total Citations. ^1^ Impact Factor from the 2019 Journal Citation Reports.

## Data Availability

The datasets analyzed in the present study are available from the corresponding author on reasonable request.
